# Right Atrial Septal Lead Enhances the Favorable Effects of the Adaptive Cardiac Resynchronization Therapy Algorithm

**DOI:** 10.1016/j.jacasi.2024.01.010

**Published:** 2024-04-03

**Authors:** Yuichiro Miyazaki, Kohei Ishibashi, Nobuhiko Ueda, Toshihiro Nakamura, Satoshi Oka, Akinori Wakamiya, Kenzaburo Nakajima, Mitsuru Wada, Takeshi Aiba, Kengo Kusano

**Affiliations:** a Department of Cardiovascular Medicine, National Cerebral and Cardiovascular Center, Suita, Japan; b Department of Advanced Cardiovascular Medicine, Graduate School of Medical Sciences, Kumamoto University, Kumamoto, Japan

**Keywords:** adaptive CRT, left ventricular pacing, responder, right atrial septal pacing

## Abstract

The adaptive cardiac resynchronization therapy (CRT) algorithm provides synchronized left ventricular pacing (sLVP). However, ensuring a high sLVP rate is challenging. We assessed the association between the sLVP rate and pacing sites in the right atrium. We evaluated 71 patients who underwent CRT and in whom the adaptive CRT algorithm was applied (53 men; mean age, 66 ± 14 years; median follow-up period, 301 days; IQR: 212-596 days). The atrial pacing leads were positioned in the right atrial (RA) septum in 17 patients (septal group) and in the RA appendage in 54 patients (RA appendage group), with significantly higher sLVP rates in the septal group compared with the RA appendage group (81% ± 30% vs 63% ± 37%; *P =* 0.045). In patients with first-degree atrioventricular blocks, the sLVP rates tended to be higher in the septal group. Therefore, RA septal pacing increased sLVP rates in patients undergoing CRT.

Cardiac resynchronization therapy (CRT) is an established treatment for patients with heart failure, reduced left ventricular ejection fraction, QRS prolongation, and left bundle branch block (LBBB).^1^^,^^2^ However, one-third of patients do not achieve improvements in cardiac function and/or clinical prognosis after CRT device implantation.^3^ The adaptive CRT (aCRT) algorithm was developed to continuously optimize atrioventricular (AV) and interventricular delays based on the heart rate and intrinsic AV conduction.^4^ Notably, the aCRT algorithm uses right ventricular intrinsic conduction, which can provide synchronized left ventricular pacing (sLVP) to create fusion beats with intrinsic conduction. Compared with biventricular pacing, sLVP is associated with lower risks of heart failure, hospitalization, cardiac death, and atrial fibrillation;^5^^,^^6^ however, achieving a high sLVP rate is difficult in some patients, especially those with prolonged PR intervals.^5^ Although a case series has demonstrated that the sLVP rate was increased by right atrial (RA) septal pacing in patients with prolonged PR intervals,^7^ to what extent RA septal pacing actually affects sLVP remains unknown. Therefore, this study aimed to identify the effective lead positions in the right atrium to secure a high sLVP rate.

## Methods

### Study population

This retrospective study included all consecutive patients who underwent CRT device implantation featuring the aCRT algorithm (Medtronic Inc) between January 1, 2017, and December 31, 2021, in a single center. The aCRT pacing mode was activated immediately after implantation. Patients with CRT devices incapable of implementing the sLVP mode and those with persistent atrial fibrillation, AV block, and an extremely prolonged AV interval (ie, AV intervals of >220 ms during atrial sensing or >270 ms during atrial pacing) were excluded.

### Ethics declaration

This study was conducted in accordance with the principles outlined in the Declaration of Helsinki and was approved by the ethics committee of our institution (M26-150). This retrospective study analyzed anonymous data generated after patients agreed to undergo the treatment; hence, the requirement for obtaining informed consent was waived, and patients were given the option to be excluded from the study using an opt-out approach.

### Device implantation

**Figure 1:**
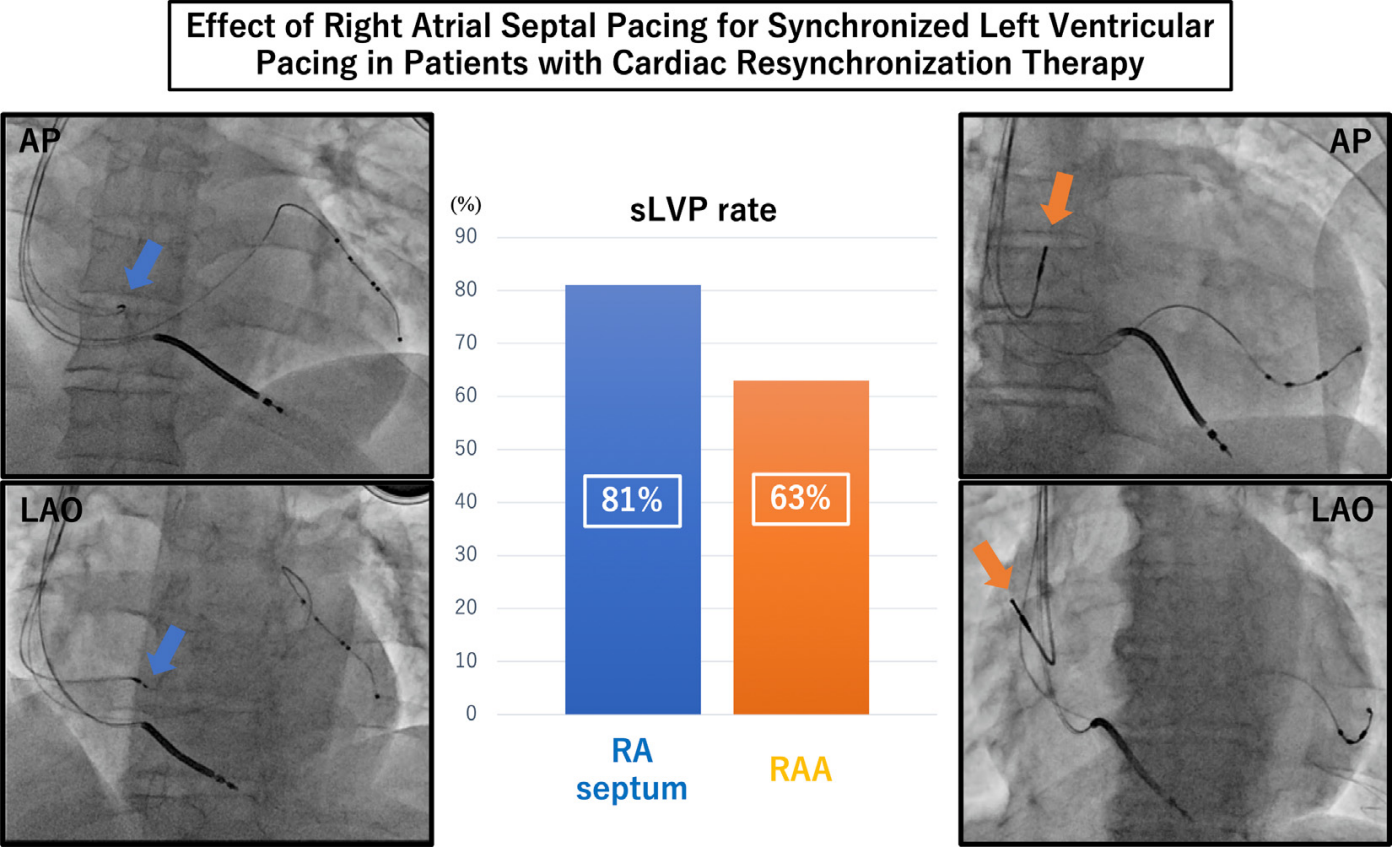
Relationship Between RA Lead Position and sLVP Rate (Left) The RA lead is positioned in the RA septum (blue arrow). (Right) The RA lead is positioned in the RAA (orange arrow). (Center) The sLVP rate was significantly higher in the septal group than in the RAA group (81% ± 30% vs 63% ± 37%; *P =* 0.045). RA = right atrial; RAA = right atrial appendage; sLVP = synchronized left ventricular pacing.

The CRT devices were implanted transvenously following a standardized procedure. A right ventricular lead was positioned in the right ventricular septal apex, and a left ventricular lead was implanted in a suitable branch of the coronary vein. Such positioning was determined based on phrenic nerve simulation avoidance, pacing threshold, and impedance. Specifically, the RA lead was placed either at the RA appendage (RAA) or septum (Figure 1), and a delivery sheath system (C315 delivery catheter C315HIS, Medtronic) was used to insert the RA lead (SelectSecure lead 3830-69, Medtronic) into the RA septum. The RA lead position (at the RAA or RA septum) was determined by the attending surgeon depending on the condition of the right atrium and confirmed using fluoroscopy of the anteroposterior or right anterior oblique and left anterior oblique views.

### Statistical analyses

Data were analyzed using the JMP software version 11.2.01 (SAS Institute). Continuous variables were expressed as mean ± SD and compared using the Student’s *t*-test. Categorical variables were compared using Fisher exact test. The strength of the association between the PR interval and the sLVP rate was quantified using Pearson’s correlation coefficient. Values of *P* < 0.05 were considered statistically significant.

## Results

### Patient characteristics

Among the 258 consecutive patients who underwent CRT device implantation between January 2017 and December 2021, we evaluated 71 patients who underwent CRT without considerably prolonged AV conduction and in whom the aCRT algorithm was applied. The patients’ mean age was 66 ± 14 years, and 53 (75%) were men. The median follow-up period was 301 days (IQR: 12-596 days). The patient characteristics are shown in Table 1. Twenty-three patients (32%) had ischemic cardiomyopathy, and the mean left ventricular ejection fraction was 23% ± 7%. The mean PR and QRS intervals were 193 ± 32 ms and 156 ± 25 ms, respectively, and 35 patients (49%) presented with LBBB. Patients with a low sLVP rate (sLVP of <50%) tended to have complete right bundle branch blocks more often than those with high sLVP rates (sLVP of >50%; 20.0% vs 3.9%; *P =* 0.049). At the 6-month follow-up after CRT implantation, the mean sLVP rate was 67% ± 36%. The pacing rate at 12 months after CRT implantation was 66% ± 39%.

**Table 1:** Characteristics of the RA Septal Pacing and RAA Pacing Groups

	All Patients (N = 71)	RA Septal Pacing (n = 17)	RAA Pacing (n = 54)	*P* Value
Age, y	66 ± 14	69 ± 12	65 ± 14	0.194
Male	53 (75)	14 (82)	39 (72)	0.530
Body weight, kg	60 ± 13	58 ± 11	61 ± 13	0.408
Ischemic cardiomyopathy	23 (32)	7 (41)	16 (30)	0.388
Hypertension	27 (38)	8 (47)	19 (35)	0.403
Diabetes mellitus	21 (30)	8 (47)	13 (24)	0.125
Chronic kidney disease	38 (54)	10 (63)	28 (52)	0.571
LVEF, %	23 ± 7	25 ± 7	23 ± 7	0.233
LVESV, mL	179 ± 82	149 ± 50	184 ± 86	0.133
PR interval, ms	193 ± 32	198 ± 37	192 ± 31	0.532
QRS interval, ms	156 ± 25	163 ± 20	154 ± 26	0.138
LBBB	35 (49)	10 (59)	25 (46)	0.414
sLVP, %	67 ± 36	81 ± 31	63 ± 37	0.045
Atrial lead parameter at implantation				
A-wave amplitude, mV		2.1 ± 1.1	2.9 ±1.3	0.030
Pacing threshold, V/0.4 ms		0.8 ± 0.4	0.7 ± 0.5	0.442
Impedance, Ω		665 ± 181	642 ± 181	0.656

Values are mean ± SD or n (%).

LBBB = left bundle branch block; LVEF = left ventricular ejection fraction; LVESV = left ventricular end-systolic volume; RA = right atrial; RAA = right atrial appendage; sLVP = synchronized left ventricular pacing.

### Clinical outcomes of RA septal pacing

Among the 71 included patients, RA septal lead implantation was attempted in 19 and successful in 17 patients. The reason for RA septal lead implantation failure was a considerably high threshold (RA septum thresholds: 3.0 V at 0.4 ms and 2.0 V at 0.4 ms). In the patients with an RA septal lead, the RA lead was implanted mainly around the coronary sinus ostium. The most frequently implanted site was the posterior margin of the coronary sinus ostium. The sLVP rate was significantly higher in patients with an RA septal lead than in those with an RAA lead (81% ± 30% vs 63% ± 37%; *P =* 0.045) (Figure 1), although no significant difference was observed in the PR interval and LBBB frequency (198 ± 36 ms vs 192 ± 31 ms; *P =* 0.532; 59% vs 46%; *P =* 0.414, respectively). At atrial lead implantation, the pacing threshold and impedance were similar between the two pacing sites. However, the A-wave amplitude at RAA was higher than that of the RA septum (Table 1). The PR interval and sLVP rate showed a negative correlation in patients with RAA pacing. However, the correlation was not significant in patients with RA septal pacing (RAA group: r = −0.588, *P* < 0.001; RA septal pacing group: r = −0.478; *P =* 0.052). In patients with first-degree AV blocks, the sLVP rates tended to be higher in patients with RA septal pacing (RAA pacing vs RA septal pacing, 41% ± 38% vs 64% ± 14%; *P =* 0.182). Additionally, regardless of the RA pacing rate, patients with an RA septal lead showed a high sLVP rate (RA pacing rate >50% vs <50%, 76% ± 37% vs 85% ± 26%; *P =* 0.566). In patients with RA septal pacing, no significant difference was found in the percentages of atrial pacing between the high and low sLVP groups.

## Discussion

This study confirmed that patients with an RA septal lead had a higher sLVP rate than those with an RAA lead.

### Favorable effects of RA septal pacing compared with that of sLVP

RA septal pacing has been shown to result in a shorter AV interval than RAA pacing.^7^ This result was highlighted in a case report in which sLVP rates were increased by RA septal pacing in patients with extremely prolonged PR intervals (424 ms). Consistent with our findings, some patients with RA septal pacing, especially those with prolonged PR intervals, had higher sLVP rates. Advanced electroanatomical remodeling of the left atrium has been associated with a prolonged PR interval.^8^ This remodeling seems to simultaneously affect the right atrium, suggesting that damage to the intra-atrial conduction may manifest as prolonged PR intervals. Therefore, in patients with intra-atrial conduction delays, RA septal pacing may prevent intra-atrial conduction delays and lead to a shortened AV interval. Thus, positioning the RA pacing lead within the RA septum of patients with prolonged PR intervals may be beneficial to increase their sLVP rates. However, RA septal pacing does not shorten the AV interval at all sites in the septum. Therefore, seeking locations that avoid intra-atrial conduction delay is necessary.

### Association between sLVP and other factors such as PR interval

Although the aCRT uses an automatic adjustment algorithm for AV and interventricular delays based on the results of frequent AV conduction evaluations, the sLVP is often used to assess normal AV conduction.^9^ Moreover, low sLVP rates have been frequently observed in men, patients without LBBB, and those with prolonged AV intervals,^10^ and such factors have been suggested to prevent sLVP. However, in this study, LBBB and long PR intervals were not associated with low sLVP rates, which we believe is due to sLVP remaining high, even in patients with prolonged PR intervals because of RA septal pacing. Although repairing a type of bundle branch block may be challenging, correcting the AV interval to increase the sLVP rate by changing the RA pacing position is possible.

### Study limitations

Regardless of the merits mentioned, this study had some limitations. First, this was a retrospective single-center study and, thus, requires that its results be confirmed further in prospective multicenter studies. Second, the sample size was relatively small, decreasing the statistical strength of our findings. Hence, future studies should consider using a larger sample size in a multicenter facility to overcome this limitation. Third, we believe that RA septal pacing should be used in patients with first-degree AV blocks. However, the AV interval of some patients could not be shortened, which poses a significant limitation. Fourth, RV lead position or right bundle branch block can affect the sLVP rate. The influence of the ventricular conduction system on the sLVP, such as delayed conduction to the RA lead, requires future assessments.

## Conclusions

Right septal pacing increases the sLVP rate in patients undergoing CRT.

## Funding Support and Author Disclosures

Drs Kusano and Ueda have received honoraria for lectures from Medtronic Japan Co, Ltd. Dr Ishibashi has received honoraria for lectures from Medtronic Japan Co, Ltd, and Biotronik Japan Inc. All other authors have reported that they have no relationships relevant to the contents of this paper to disclose.
